# The Interaction of 2D Materials With Circularly Polarized Light

**DOI:** 10.1002/advs.202206191

**Published:** 2023-01-25

**Authors:** Rong Rong, Ying Liu, Xuchen Nie, Wei Zhang, Zhuhua Zhang, Yanpeng Liu, Wanlin Guo

**Affiliations:** ^1^ Key Laboratory for Intelligent Nano Materials and Devices of the Ministry of Education State Key Laboratory of Mechanics and Control of Mechanical Structures and Institute for Frontier Science Nanjing University of Aeronautics and Astronautics Nanjing 210016 China

**Keywords:** 2D materials, chiral light–matter interaction, circularly polarized light, light‐induced phenomena

## Abstract

2D materials (2DMs), due to spin‐valley locking degree of freedom, exhibit strongly bound exciton and chiral optical selection rules and become promising material candidates for optoelectronic and spin/valleytronic devices. Over the last decade, the manifesting of 2D materials by circularly polarized lights expedites tremendous fascinating phenomena, such as valley/exciton Hall effect, Moiré exciton, optical Stark effect, circular dichroism, circularly polarized photoluminescence, and spintronic property. In this review, recent advance in the interaction of circularly polarized light with 2D materials covering from graphene, black phosphorous, transition metal dichalcogenides, van der Waals heterostructures as well as small proportion of quasi‐2D perovskites and topological materials, is overviewed. The confronted challenges and theoretical and experimental opportunities are also discussed, attempting to accelerate the prosperity of chiral light‐2DMs interactions.

## Graphical abstract

The interaction between 2D materials and circularly polarized light (CPL) provides an attractive control knob for investigating emergent exotic phenomena, especially for chiral‐electronics, photonics, and optoelectronics effects. This review summarizes exciting chiral light‐induced phenomena in representative 2DMs, covering from distinct interaction mechanisms, advances, faced challenges and perspectives, attempting to boost tremendous applications in future optoelectronics, spintronics, and valleytronics.

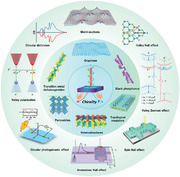

## 1 Introduction

2D materials (2DMs), due to atomic thickness and confinement effects, exhibit fascinating properties that are absent in their bulk counterparts and have attracted much attention in the multidisciplinary research community.^[^ ^1^, ^2^, ^3^, ^4^, ^5^ ^]^ Moreover, the high aspect ratio and planar/flexible morphology offer 2DMs great opportunities toward electronic and optoelectronic applications.^[^ ^6^, ^7^, ^8^, ^9^, ^10^ ^]^ Stimulated by the success of graphene, a vast library of 2D materials comprising transition metal dichalcogenides (TMDs), black phosphorus (BP), van der Waals (vdWs) heterostructures, perovskites, and even layered topological insulators (TIs) emerges with ever‐increasing material members and categories.^[^ ^11^, ^12^, ^13^, ^14^, ^15^, ^16^, ^17^, ^18^ ^]^ In comparison with conventional metal or semiconducting materials, electrons in 2DMs possess intrinsic degree of freedom: charge, spin, and even valley in the premise of time‐reversal symmetry breaking.^[^ ^19^ ^]^ Thanks to the versatile material platforms and additional valley degree of freedom, intriguing phenomena, for example, anomalous Hall Effect, circular photogalvanic effect, and valley polarization, have been reported theoretically and experimentally in succession.^[^ ^20^, ^21^ ^]^ In particular, in combination of two structural dimensionality, different external stimuli play an essential role in probing and manifesting the physical properties of 2DMs, such as electrical gating, static magnetic fields, cryo‐environments, and laser excitations. Out of them, the phenomenological adventure of 2DMs physics by optical means boosts an explosion of fundamental research because of tunable light polarization and light–matter interactions.^[^ ^22^, ^23^ ^]^

The superiority of light residue in its controllable degree of freedoms includes angular momentum, energy, and linear momentum.^[^ ^24^, ^25^ ^]^ The angular momentum of light is determined by its helicity (*σ*) and *σ* = +1/−1 representing right (RCP) and left circular polarization (LCP), respectively. Profiting from the helicity, the circularly polarized light is capable to excite or manipulate emerging physical properties of 2DMs and unpredictable brand‐new physics are excavated, especially for chiral‐electronics, photonics, and optoelectronics.^[^ ^26^, ^27^ ^]^ Take monolayer TMDs as example, energy levels at K and K′ valleys antisymmetric spin states might degenerate owing to the broken inversion symmetry or strong spin–orbit coupling (SOC) from heavy transition metal atoms. In accordance to valley‐contrasting optical selection rules, the interband transition at distinct K (or K′) valley selectively couples with circularly polarized light (CPL) with certain helicity, harboring assorted valleytronic phenomena including valley polarization, valley Hall effect, and valley Zeeman effect.^[^ ^28^ ^]^ Stimulated by this, CPL was successively implemented into other 2D materials and their vdWs heterostructures, quasi‐2D TIs, and perovskite systems. As a result, CPL‐induced spin Hall effect, photonic spin Hall effect, circular photogalvanic effect (CPGE), and circular dichroism (CD) effect were recently predicted or observed.^[^ ^29^, ^30^ ^]^ Although the research competition on CPL‐2DMs interaction reaches an impressive level, few reviews exist seemingly relevant to this hot topic but they mainly focus on the circularly polarized luminescence through chiral luminophores,^[^ ^31^, ^32^, ^33^, ^34^ ^]^ or fixed 2D materials interacting with various lights.^[^ ^35^, ^36^ ^]^ In this regard, a thorough understanding and systematical overview of the interaction between circularly polarized lights and 2DMs is still lacking, as well as the light–matter interaction mechanisms and advances.

**Figure 1:**
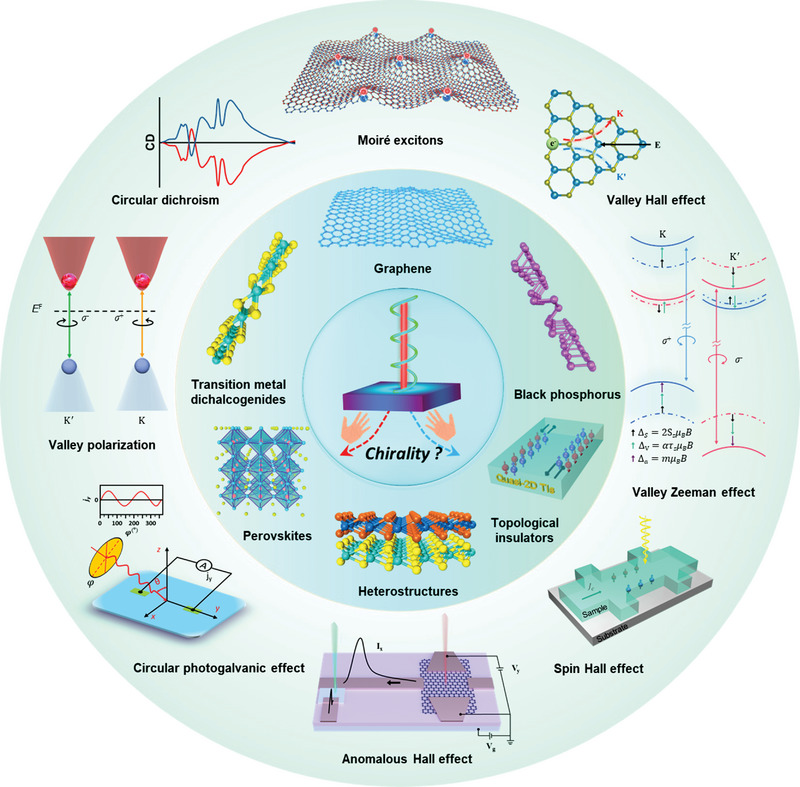
The scope of this review is mainly chiral light‐induced emerging phenomena on the basis of graphene, black phosphorus, transition metal dichalcogenides, heterostructures, perovskites, and topological insulators.

In this review, we discuss the recent process on the exotic phenomenon of 2DMs probed and manipulated by circularly polarized light on the basis of graphene, black phosphorus, transition metal dichalcogenides, van der Waals heterostructures, perovskites, and layered topological insulators. For each host material (**Figure** **1**), when their constituent structures and intrinsic properties are introduced, we focus on emerging electronic and optoelectronic effects that interact with circularly polarized light and synchronize with the discussion of underlying physical mechanisms. Last, faced challenges and perspectives toward spintronic, valleytronics, and memory applications are also presented.

## 2 Graphene

The initial research of 2DMs originates from the discovery of graphene in 2004.^[^ ^37^ ^]^ Graphene, a single‐layer of carbon atoms arranged in honeycomb lattice, possesses a relativistic massless dispersion and became a research hotspot in multidisciplinary research community.^[^ ^38^, ^39^ ^]^ Moreover, chiral features in the electronic wavefunctions offer graphene intriguing physical, electronic, and optical properties. After breaking the inversion symmetry or creating a bandgap in graphene,^[^ ^40^, ^41^ ^]^ striking properties beyond traditional conductive materials, such as valley polarization,^[^ ^42^, ^43^ ^]^ anomalous Hall effect,^[^ ^44^, ^45^ ^]^ photonic spin Hall effect^[^ ^46^ ^]^ and circular photogalvanic effect^[^ ^47^, ^48^ ^]^ were predicted or experimentally verified. In this section, we attempt to overview the important breakthroughs in CPL–graphene interactions.

### 2.1 Valley Polarization

Graphene, due to two inequivalent sub‐lattices, has degenerated and polarized K and K’ valleys in Brillouin zone in the premise of breaking the inversion symmetry and/or time‐reversal symmetry. In this scenario, the inter‐band transitions in the vicinity of the K (K′) point couple exclusively to right (left) circularly polarized light in accordance to valley‐contrasting optical selection rules.^[^ ^42^ ^]^ This coupling between valley excitons and photons with proper helicity provides a protocol to control the valley polarization. However, the valance band and conduction band of pristine graphene meet at Γ point, leading to valley polarization (VP) that is usually invisible. To introduce a bandgap, to our best knowledge, three routines were reported to date. The first one is transferring graphene onto hexagonal boron nitride^[^ ^41^ ^]^ or silicon carbide.^[^ ^49^ ^]^ The staggered sublattice potential from the substrate coupling breaks the inversion symmetry of graphene and opens a bandgap up to 0.5 eV. Besides, electrostatic doping of biased bilayer graphene through gating was proven to be an alternative approach to break symmetry with a sizable bandgap.^[^ ^50^, ^51^, ^52^ ^]^ Last, a combination of two counter‐rotating circularly polarized fields (bi‐circular electric field) was reported to successfully break the inversion symmetry of graphene and then control the relative excitation probabilities of K and K′ valleys.^[^ ^53^ ^]^ Among different bandgap‐opening methods, the superposing of graphene onto boron nitride was predicted to break the inversion symmetry and create a small bandgap (less than tens of meV).^[^ ^41^ ^]^ Similarly, the epitaxial grown graphene on silicon carbide exhibited a larger bandgap (≈0.5 eV) but high temperatures over 1000 °C required for sample growing limit their applications.^[^ ^49^ ^]^ Second, the electrostatic gating of bilayer graphene was found to achieve a bandgap covering the electromagnetic ranges from zero to mid‐infrared. This approach realizes a sizeable bandgap and avoids uncontrolled chemical contaminations, but the tedious architecture adds to the difficulty in device fabrication.^[^ ^40^ ^]^ Last, the inversion symmetry of graphene could be broken by bi‐circular fields.^[^ ^53^ ^]^ It offers a simple and ultrafast route to open a bangap but the premature experimental setup restricts this all‐optical method in the theoretical stage.

The advent of breaking graphene symmetry has enabled fantastic process of valley polarization toward valley‐dependent electronics and optoelectronics. However, the influences of possible quantized landau levels, layer number, and orientation angles in graphene stacks to valley polarization lagged unknown.^[^ ^54^, ^55^ ^]^ Moreover, as aforementioned, valley polarization of graphene requires extreme conditions and complicated combinations of several manipulation technologies. Therefore, the majority of graphene valley polarization studies are conducted theoretically. Experimental tuning and observations remain challenges at the current stage and more efforts are urgently needed to excavate this treasurable field.

### 2.2 Light‐Induced Anomalous Hall Effect

It is recognized that external magnetic fields are essentially required to drive electrons in traditional Hall effect. On the contrary, the anomalous Hall effect (AHE) refers to the appearance of a noticeable spontaneous Hall current in ferromagnet materials in response to a source‐drain current without external magnetic fields.^[^ ^56^ ^]^ Since the beginning of this century, the intrinsic AHE mechanism was reformulated in terms of the Berry phase and topological properties of electronic wave functions.^[^ ^57^, ^58^ ^]^ For an asymmetric graphene system, the main origin of intrinsic AHE was ascribed to the Berry curvature that behaves as an artificial gauge field in momentum space. In the aspect of topological graphene, CPL driving was proposed to be an effective approach in inducing AHE in graphene.^[^ ^59^, ^60^ ^]^

**Figure 2:**
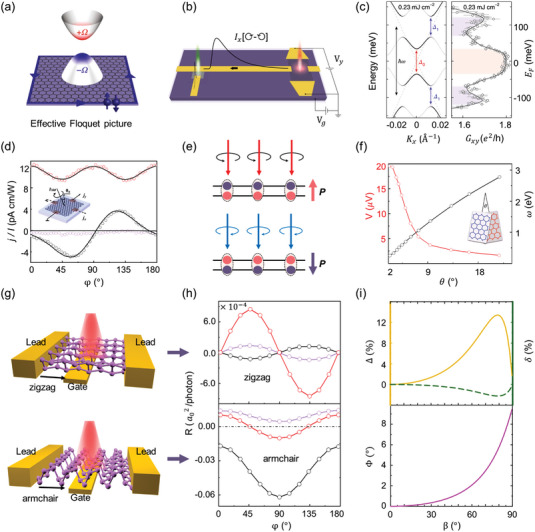
CPL‐induced phenomena in graphene and black phosphorus. a) Light‐induced topological Floquet bands in graphene. b) Device schematic for detecting anomalous Hall currents. c) Left panel: simulated band structures under laser pulses (fluency up to 0.23 mJ cm^−2^) using Floquet theory. Right panel: Anomalous Hall conductance (*G_xy_*) as a function of *E*_F_. Reproduced with permission.^[^ ^44^ ^]^ Copyright 2019, Springer Nature. d) Photocurrent versus the angle (*φ*) curves. Red and black circles show the longitudinal (*j_x_*) and transverse (*j_y_*) photocurrents measured at oblique incidence (*θ*_0_ = −30°), respectively. No photocurrent (purple circles) was generated at normal incidence (*θ*_0_ = 0°). The inset shows the device architecture. Reproduced with permission.^[^ ^47^ ^]^ Copyright 2011, American Physical Society. e) Proposed mechanism of the Layer‐CPGE. Pink and dark purple disks represent negative and positive charges, respectively. f) The peak position (black) and corresponding potential difference (red). Reproduced with permission.^[^ ^48^ ^]^ Copyright 2020, American Physical Society. g) Device schematic of BP‐based photodetector along the zigzag and armchair directions, respectively. h) Photocurrents of BP device versus the helicity of CPL along zigzag and armchair directions. Reproduced with permission.^[^ ^62^ ^]^ Copyright 2018, RSC Pub. i) Performance of an unstructured BP sheet in circular dichroism (yellow line), circular conversion dichroism (green dashed line), and circular birefringence (purple line) for varied incident angles (*β*) with fixed frequency at 6 THz. Reproduced with permission.^[^ ^63^ ^]^ Copyright 2019, Optical Society of America.

The nonlinearity of CPL was theoretically demonstrated to create a topological bandgap (renamed the Floquet method) in the Dirac cone.^[^ ^61^ ^]^ **Figure** **2**a illustrates the non‐zero Berry curvature distribution and topologically protected edge states that induce robust AHE. Motivated by this prediction, Mclver et al.^[^ ^44^ ^]^ experimentally detected the light‐induced AHE in monolayer graphene device via a laser‐triggered photoconductive switch under femtosecond CPL pulses (Figure 2b). In their experiments, the polarity of *I_x_* [⟳–⟲] signal (the current difference induced by RCP and LCP lights) reversed when switching the CPL helicity or the source‐drain voltage (*V_y_*). The amplitude of *I_x_* showed a linear dependence on *V_y_*, revealing the CPL‐induced anomalous Hall currents. In addition, the AHE was further proved to be regulated by the gate potential that determines the Fermi level. In the topological Floquet bands (Figure 2c), a conductance plateau (width ≈ 60 meV, 1.8 ± 0.4 *e* ^2^ *h* ^−1^) appeared at Fermi level |*E*_F_| ≲ 30 meV, suggesting the existence of a bandgap and AHE. Soon after, Sato et al.^[^ ^45^ ^]^ explored the microscopic origin of the light‐induced AHE by taking relaxed quantum Liouville equations into account. The simulated result confirmed that the light‐induced AHE originated from both the asymmetric distribution of photocarriers in the topological Floquet bands and the anomalous velocity from Berry curvature.

### 2.3 Photonic Spin Hall Effect

Photonic spin Hall effect (PSHE), a photonic analogy of spin Hall effect in the electronic systems, originates from spin–orbit interaction with light and manifests itself as a transverse spin‐dependent splitting through light–matter interactions.^[^ ^64^ ^]^ To be more specific, PSHE refers to the in‐plane and/or transverse spin‐dependent shifts from the left and right circularly polarized components when a spatially‐confined light beam undergoes reflection or transmission at an interface between two materials with different refractive indices. Initially, PSHE was theoretically and experimentally verified in the scenario that photons are reflected or refracted on the air–glass interface.^[^ ^65^ ^]^ After that, how to control the spin‐dependent splitting of light in an elegant way has become one of the hot topics in the research community of 2DMs.

To our best knowledge, external fields or rationale structural models are the prevalent ways to regulate PSHE in an elegant manner. The PSHE modulations in graphene systems were reported by magnetic fields in accordance with the intense magneto‐optical response of graphene.^[^ ^66^, ^67^ ^]^ By regulating the rotation angle of incident light, external magnetic fields effectively modulated the asymmetric PSHE shifts. The author further proposed the applications in barcode encryption by employing the transverse shifts for LCP and RCP components as binary digits.^[^ ^66^ ^]^ Second, introducing a light beam reflected at the glass–air interface pre‐coated with monolayer graphene has been theoretically reported for dynamically controlling the PSHE. The in‐plane spin shifts can be significantly adjusted by tuning graphene Fermi energy.^[^ ^68^ ^]^

### 2.4 Circular Photogalvanic Effect

In the absence of inversion symmetry, the CPL‐excited electrons distributed in graphene conduction bands are unbalanced and consequently produce a persistent photocurrent even with no external bias. This phenomenon is defined as the circular photogalvanic effect (CPGE). By supposing graphene on silicon carbide to break the inversion symmetry, Jiang et al. reported the helicity‐driven photocurrents with no source‐drain bias.^[^ ^47^ ^]^ Figure 2d shows the transversal photocurrent (*j_y_*) under illumination at oblique incidence. The sign of *j_y_* reversed when reversing the CPL helicity and tuning the CPL frequency. According to their phenomenological and microscopic analysis, the helicity‐driven photocurrent was attributed to the interplay of CPGE and circularly alternating current Hall effect.

Twisted bilayer graphene that naturally lacks any mirror plane and inversion center is another ideal platform for probing and even tuning CPGE on‐demand. For instance, Gao et al.^[^ ^48^ ^]^ proposed a general theory of layer circular photogalvanic effect (Layer‐CPGE) in chiral twisted bilayer graphene. In their study, the CPL excitation of twisted bilayer graphene at normal incidence resulted in a static out‐of‐plane dipole moment. As shown in Figure 2e, the direction of dipole moment was highly correlated with CPL helicity. Moreover, the Layer‐CPGE coefficient became highly tunable with the twist angle and photo‐energy (Figure 2f). To be more specific, the resonant excitation frequency (*ω*) of light varies from visible to infrared by decreasing the twist angle from 22° to 2°. As the Layer‐CPGE coefficient (*β*) is proportional to 1/*ω* ^2^, the induced voltage difference increases significantly with decreasing twisting angle and achieves a peak value of ≈20 µV at the twist angle ≈2°. In addition, the Layer‐CPGE signal can be further enhanced in multilayer chiral stacked structures. Although the origin of Layer‐CPGE phenomenon is still theoretical, this study provides an avenue to design optical materials with desired chirality.

## 3 Black Phosphorus

Black phosphorus (BP), phosphorus atom covalently bonded with three adjacent atoms in a puckered structure, exhibits layer‐dependent direct bandgaps (from 0.3 eV in the bulk to 2.0 eV in monolayer) and shows great promise in light–matter interactions.^[^ ^69^ ^]^ Moreover, BP crystal exhibits strong in‐plane anisotropy in electronic and optical properties, including anisotropic optical absorption and photoluminescence.^[^ ^70^ ^]^ Profiting from these, BP became a versatile platform for investigating circular photogalvanic effect, optical activity, and photonic spin Hall effect once the inversion symmetry was broken.

### 3.1 Circular Photogalvanic Effect

The crystal structure of BP belongs to *D*_2h_ point group, giving BP mirror reflection symmetry and inversion symmetry. To break this inherent symmetry, several methods have been theoretically predicted via density functional theory within the non‐equilibrium Green's function formalism for inducing CPGE as follow.^[^ ^71^, ^72^ ^]^

One feasible solution is to break the space inversion symmetry of BP by introducing dopant (sulfur atoms) into BP layer as well as induced electrical fields.^[^ ^62^, ^73^ ^]^ For the latter case, Li et al.^[^ ^62^ ^]^ broke the inversion symmetry (Figure 2g, *V*_g_ = 1.0 V) in few‐layer BP device by CPL illumination and electrostatic gating resulting from the redistribution of effective potentials. The induced photocurrents were found to be sine (cosine) dependent on the CPL helicity (Figure 2h). As a supplement, the authors further superimposed phosphorene onto blue phosphorene layer to induce *C*_s_ lattice symmetry by sacrificing the inversion symmetry. Remarkable anisotropic photocurrents confirmed the CPL‐induced CPEG in BP‐based heterostructure. As an external bias voltage is not essential to drive photo‐excited electrons, CPGE is of great importance to suppress dark current and reduce the signal‐to‐noise ratio for BP‐based optoelectronic devices. Therefore, more efforts are highly encouraged into investigating CPGE for BP‐based photodetectors, in combination with relatively mature theoretical predictions.

### 3.2 Optical Activity

Optical activity denotes the ability to control light polarization and could be divided into circular dichroism (CD) and circular birefringence effects. Circular dichroism represents the differential transmission (or reflection/absorption) between LCP and RCP lights, while circular birefringence refers the ability in rotating the polarization plane of light. Similar to afore‐mentioned CPEG, the premise for observing optical activity lies in the mirror asymmetry (chirality) or broken time‐reversal symmetry. Recently, BP monolayer was theoretically proved to exhibit robust optical activity under the oblique incidence of circularly polarized light.^[^ ^63^ ^]^ The BP chirality was induced by the mutual orientation of incident beam and BP plane. As shown in Figure 2i, the circular dichroism (*∆*) elevated with increasing the incidence angle (*β*), reached the maximum of 13.41% at 79° and then rapidly dropped to 0 at 90°. Meanwhile, the circular birefringence (*Φ*) increased when the incident angle changed from 0° to 89.9°. In another report, enhanced optical activity with the reflected light rotation about 90° was reported in BP thin film that works as a natural hyperbolic metasurface, making BP films promising for polarization applications and modern flat optics.^[^ ^74^ ^]^ External magnetic fields along normal incidence direction broke the time‐reversal symmetry and obtained optical activity in monolayer BP. The optical activity was found to vary monotonically with the magnetic fields. For instance, when the magnetic field was increased from 4 T to 10 T, the circular dichroism almost linearly increased from 0.043 to 0.102 and the circular birefringence could be tuned from ≈ −3 to ≈ −8 concurrently. In a nutshell, these studies manifesting BP optical activities ignite the potential applications toward polarization optics and stereochemistry.^[^ ^75^ ^]^

### 3.3 Photonic Spin Hall Effect

Theoretical predictions reveal that the asymmetric spin splitting and the photonic spin Hall effect (PSHE) may occur in BP because of its anisotropic nature.^[^ ^76^ ^]^ In the aspect of asymmetric spin splitting, spin‐independent and spin‐dependent elements of the centroid displacements drive the RCP and LCP components oppositely. PSHE in BP were pioneerly verified by Zhang and coworkers, the transverse and in‐plane spin‐dependent splitting lay in BP surface. In addition, the spin‐dependent shifts were further found to be sensitive to the orientation of optical axis, doping concentration, and interband transitions.^[^ ^77^ ^]^ One step further, in the present of magnetic fields, both the transverse and in‐plane spin‐dependent shifts were quantized and oscillated due to the Landau levels splitting in BP monolayer.^[^ ^78^ ^]^ However, the inmost mechanism of the quantized and enhanced spin shifts in both transverse and in‐plane directions remain debated.

Originating from in‐plane anisotropy, the spin‐independent shifts in BP contain Goos–Hänchen and Imbert–Fedorov shifts that lead to asymmetric spin splittings.^[^ ^76^ ^]^ Meanwhile, the asymmetric spin splitting could be further enhanced by the incident orbital angular momentum and bias voltages, potential for applications in four‐channel barcode encoding. In the frame of PSHE in BP, a highly‐tunable terahertz sensor was theoretically proposed with the sensitivity of spin‐dependent shift up to 2804 mm/RIU and a refractive index resolution of ≈10^−8^ RIU, promising for applications in chemical sensing and biosensing.^[^ ^79^ ^]^

## 4 Transition Metal Dichalcogenides

**Figure 3:**
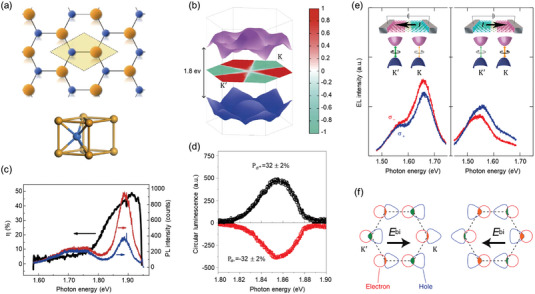
Valley polarization in mono‐ and multi‐layer TMDs. a) Top panel: Top view of monolayer TMDs composed of transition metal atoms (blue) and chalcogen atoms (yellow). The shaded region bounded by dashed lines corresponds to one primitive cell. Bottom panel: Side view of TMDs structure. b) Valley‐selective CD of monolayer MoS_2_. The central hexagon is the Brillouin zone, colored by the degree of circular polarization. c) Circularly polarized PL and the net degree of circular polarization of monolayer MoS_2_ at 83 K. Reproduced with permission.^[^ ^81^ ^]^ Copyright 2012, Springer Nature. d) Polarization resolved PL of monolayer MoS_2_ from a HeNe laser at 1.96 eV and 10 K. Reproduced with permission.^[^ ^82^ ^]^ Copyright 2012, Springer Nature. e) Circularly polarized EL from a WSe_2_ *p–n* junction under two opposite current directions. f) Schematic illustrations of electron and hole distributions shifted by electric fields. Orange and green areas represent the electron–hole overlap for K and K′ valleys, respectively. Reproduced with permission.^[^ ^91^ ^]^ Copyright 2014, AAAS.

Transition metal dichalcogenides (TMDs) have the chemical composition of *M*‐*X*‐*M* (*MX*_2_), where *M* stands for the transition metal atoms (such as W and Mo) and *X* represents chalcogen atoms (S, Se etc). Monolayer TMDs consist of a single layer of transition metal atoms sandwiched between two chalcogen layers in the trigonal prismatic structure (**Figure** **3**a). In the hexagonal Brillouin zone, the conduction and valence band edges of TMDs located at the K and K′ points and the corresponding bandgap energy lie in near‐infrared to visible frequency range. When TMDs are thinned to a single layer, the inversion symmetry is explicitly broken and valley‐contrasting optical selection rule becomes applicable.^[^ ^80^ ^]^ In the next, we will focus on the interaction of TMDs and circularly polarized lights, including but not limited to valley polarization, valley Hall effect, valley Zeeman effect, optical Stark effect, and circular photogalvanic effect.

### 4.1 Valley Polarization

TMDs monolayers with innate inversion symmetry breaking exhibit vast possibilities for observing valley polarization, and the broken inversion symmetry allows contrasted circular dichroism in different regions of Brillouin zone and valley‐contrasting optical selection rule at high symmetry points.^[^ ^42^ ^]^ In other words, valley polarization becomes experimentally readable through polarization‐resolved photoluminescence. Generally, the degree of circular polarization is defined as,

(1) $$P=\frac{I\left(\sigma +\right)-I\left(\sigma -\right)}{I\left(\sigma +\right)+I\left(\sigma -\right)}$$

where *I*(σ±) is the intensity of left (right) circular components and used to quantitatively evaluate valley polarization. Valley polarization was first predicted via valley‐selective circular dichroism (CD) in monolayer MoS_2_ by density functional perturbation theory.^[^ ^81^ ^]^ As shown in Figure 3b, the sign of valley polarization is opposite for two valleys. Correspondingly, both circular polarization and chiral absorption selectivity were −1(+1) for K (K′) valley. To verify the simulation, circularly polarized photoluminescence (PL) of monolayer MoS_2_ showed a significant valley‐selective CD (*P* ≈ 50%, Figure 3c; ≈32%, Figure 3d).^[^ ^81^, ^82^ ^]^ Subsequently, valley polarization was enhanced to nearly 100% by improving the crystal quality or adopting h‐BN as the substrates to suppress the intervalley scattering.^[^ ^83^, ^84^, ^85^ ^]^ For these perfect circular polarization values, the steady‐state PL measurements confirm their origins from better intervalley processes with fast valley lifetime (≈1.5–10 ps) and long valley lifetime (several tens of ps) for trion emission.^[^ ^86^, ^87^, ^88^, ^89^ ^]^

For multilayer TMDs, broken inversion symmetry is also the prerequisite for investigating valley polarization. Differentiating from monolayer counterpart; however, multilayers TMDs are indirect‐gap semiconductors with the inversion symmetry. To break the inversion symmetry, out‐of‐plane electrical fields were adopted to induce valley‐contrasting optical selection rules.^[^ ^90^ ^]^ In their study, the PL polarization of bilayer MoS_2_ was continuously tuned from −15% to +15% by sweeping the gate from −80 V to +20 V. Moreover, by creating a lateral *p*–*n* junction using ion‐gel gating (Figure 3e), the circularly polarized electroluminescence has been observed in multilayer WSe_2_.^[^ ^91^ ^]^ After switching the source‐drain bias, the corresponding polarization reversed, suggesting the different shifts (Figure 3f) of electron–hole distributions of two distinct valleys under high electrical bias.

### 4.2 Valley/Exciton Hall Effect

**Figure 4:**
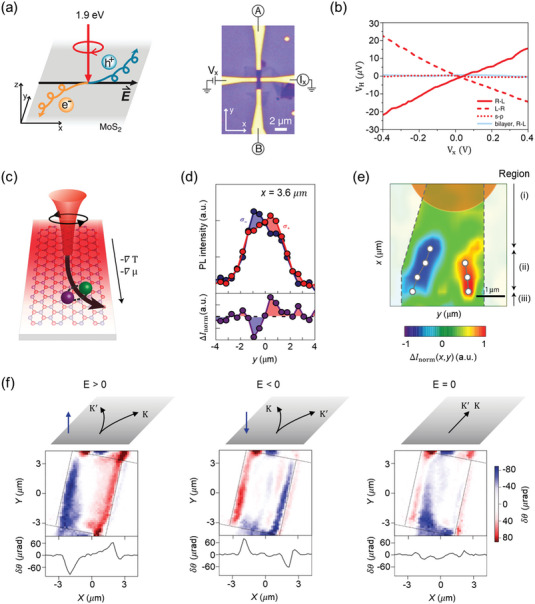
Valley Hall effect in TMDs. a) Photo‐induced AHE in monolayer MoS_2_. The absorption of CPL produces a net valley polarization (left), which creates the Hall voltage (right) in a Hall bar device. b) The helical Hall voltage of monolayer MoS_2_. Results from bilayer MoS_2_ are also revealed. Reproduced with permission.^[^ ^93^ ^]^ Copyright 2014, AAAS. c) Schematic of the exciton Hall effect. d) Top panel: cross‐sectional profiles of PL intensities at *X* = 3.6 µm. Bottom panel: normalized intensity difference (Δ*I*_norm_) as a function of *y*. e) Color plot of PL polarization. The white circles show the peak positions of Δ*I*_norm_. The Hall angle is defined by the ratio of the spin Hall current to the total dissipative current. Here, *α*_EHE_ is defined as *L_xy_*/*L_xx_*, where *L_xy_* is half of the distance between the separated peaks (*σ*+ and *σ*−) and *L_xx_* is the distance from the excitation center. Reproduced with permission.^[^ ^99^ ^]^ Copyright 2017, Springer Nature. f) Top figures: The VHE in bilayer MoS_2_ devices under different out‐of‐plane electric fields. Blue arrows indicate the direction of electric fields. Bottom figures: spatial mapping and horizontal linecut of Kerr rotation signal (*δθ*) for different *E* values. All measurements were performed at the same bias (2.5 V). Reproduced with permission.^[^ ^100^ ^]^ Copyright 2016, Springer Nature.

Valley Hall effect (VHE) refers to what a valley current generates along the transverse direction under a source–drain bias. In hexagonal 2D crystals with broken inversion symmetry, electrons at the K and K′ valleys undergo finite Berry curvature (*Ω*) with opposite signs.^[^ ^92^ ^]^ Behaving as an effective magnetic field in momentum space, the opposite Berry curvatures render the carriers from two valleys an anomalous velocity and thus drive them in opposite transverse directions, forming valley‐contrasting Hall currents. Experimentally, CPL is a necessary and effective means to break time‐reversal symmetry in monolayer TMDs toward valley polarization. In monolayer MoS_2_ device with Hall bar structure, Mak et al. demonstrated a net transverse Hall voltage (**Figure** **4**a,b) that was strongly dependent on the helicity of excitation light.^[^ ^93^ ^]^ Later, monolayer WS_2_^[^ ^94^ ^]^ and WSe_2_^[^ ^95^ ^]^ were successionally demonstrated to exhibit VHE in the same way, stimulating tremendous research efforts in exploring fresh TMDs with robust VHE.

Similar to the valley‐polarized electrons in VHE, excitons that are electron–hole pairs bound by Coulomb interaction were also tunable by circularly polarized lights.^[^ ^96^, ^97^, ^98^ ^]^ Figure 4c shows the mapping of polarization‐resolved PL intensity of monolayer MoS_2_.^[^ ^99^ ^]^ When circularly polarized light focused on one edge of the narrow device, opposite Berry curvatures produced a temperature and/or chemical potential gradient that deflected the excitons from two valleys to opposite sides of the channel. The authors clarified valley‐contrasting Hall effect of excitonic states (Figure 4d) and defined the phenomenon as the exciton Hall effect (EHE). The exciton trajectory was further visualized via real‐space mapping (Figure 4e) and then the extracted exciton Hall angle (*α*_EHE_) was up to 0.20 ± 0.09, two orders of magnitude larger than the valley Hall angle of monolayer MoS_2_.^[^ ^93^ ^]^

Next, we overview the CPL‐induced valley/exciton Hall effects in bilayer and even multilayer TMDs. The inversion symmetry of unperturbed bilayer MoS_2_ is reserved but a vertical electric field is able to break the symmetry for studying VHE. Figure 4f shows the accumulation of net valley polarizations along bilayer MoS_2_ edges by employing Kerr rotation (KR) microscopy.^[^ ^100^ ^]^ The out‐of‐plane polarization could be controlled by gate voltages. More specifically, at *V*_g_ = 20 V, clear KR signal appeared only near the two edges and the KR peak was at the level of ≈100 µrad. It is also worthy to mention that the sign of KR reversed for positive and negative gate voltages (left and middle panels, Figure 4f). The VHE observation in monolayer, bilayer, and even multilayer^[^ ^101^ ^]^ TMDs paves the way of CPL‐tunable hall devices for future valleytronics.

### 4.3 Optical Stark Effect

**Figure 5:**
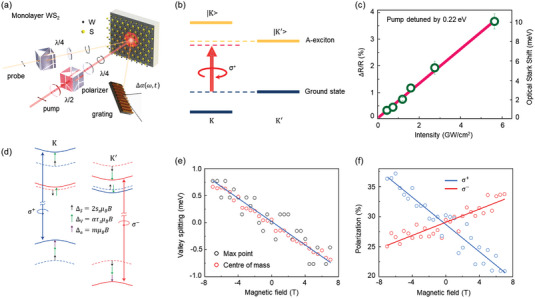
Optical Stark effect and valley Zeeman splitting in TMDs. a) Schematic of the pump–probe experiment on monolayer WS_2_. Reproduced with permission.^[^ ^103^ ^]^ Copyright 2014, Springer Nature. b) Schematic of valley‐dependent optical Stark effect with non‐resonant and left circularly polarized pump. c) The transient reflection (left axis) and corresponding energy shift (right axis) as a function of pump intensities. Reproduced with permission.^[^ ^102^ ^]^ Copyright 2014, AAAS. d) Energy level diagram of valley Zeeman effect under external magnetic field (three contributions: black for spin, green for valley, and purple for atomic orbital). e) Valley Zeeman splitting versus external magnetic fields. The “Max point” and “Center of mass” refer to the methods of extracting energies. f) Degree of PL polarization for exciton. Blue (red) indicates *σ*+ (*σ*−) excitations. Reproduced with permission.^[^ ^107^ ^]^ Copyright 2015, Springer Nature.

Locked by time‐reversal symmetry, the K and K′ valleys in monolayer TMDs are usually degenerated in energy. Lifting the valley degeneracy is essential for controlling the valley degree of freedom in the emerging valleytronics. Optical Stark effect (OSE) tunes the exciton energy levels for each valley and inevitably lifts the valley degeneracy in a valley‐selective manner. **Figure** **5**a shows the valley‐selective OSE in monolayer WSe_2_^[^ ^102^ ^]^ and WS_2_^[^ ^103^ ^]^ by pumping below‐resonance (red‐detuned) CPLs using ultrafast pump‐probe spectroscopy. Photon‐dressed (Floquet) states and non‐resonant laser field were adopted to avoid any real population excitation. The working principle is as follows (Figure 5b): after LCP pumping (*σ*+), the dressed‐ground state and the K valley A‐exciton state interacted with each other because of the Coulomb potential and hybridization, creating an energy level repulsion. After that, the ground‐state energy was shifted down while K valley A‐exciton energy was shifted up. Hence, the optical transition between ground and exciton states was shifted to a larger energy. In contrast, the states in K′ valley were not shifted by left polarized light (*σ*+); thus, lifting the energy degeneracy between K and K′ valley. Experimentally, the blue shift of K valley exciton resonance was observed only when the pump and probe pulse had the same helicity, validating their theoretical speculation. To be quantitative, the CPL‐induced energy splitting between K and K′ exciton transitions could be up to 10 and 18 meV in monolayer WSe_2_ and WS_2_ (Figure 5c), respectively. It is worth noting that the above studies are based on the hypothesis of the non‐interacting particles in the framework of the dressed two‐level system. Actually, the strong Coulomb interactions in monolayer TMDs could stimulate tightly bound excitons, trions, and inter‐valley biexcitons and other many‐body effects. Among them, biexcitonic optical Stark effects have been extensively studied recently and can be described theoretically in an expanded four‐level picture.^[^ ^104^, ^105^, ^106^ ^]^ For example, Yong et al.^[^ ^104^ ^]^ reported the biexcitonic optical Stark effects in monolayer MoSe_2_ using circularly polarized pump‐probe spectroscopy. By pumping blue‐detuned *σ*+ polarized light, an anomalous OSE was observed in addition to the ordinary OSE in K valley exciton states. The authors ascribed the anomalous behavior to the coupling between the driving photon and different excitonic states and even the many‐body intervalley biexciton states.

Optical Stark effect offers a convenient way to selectively manipulate the valleys with polarized light. Typically, the Stark shifts are detected with ultrafast pump‐probe spectroscopy that might be hampered by assorted background effects and noises. For future direction; therefore, more accurate tools and measurements are still needed to monitor optical Stark effects in TMDs.

### 4.4 Valley Zeeman Effect

In light of the valley‐dependent magnetic moment in monolayer TMDs, an external magnetic field was reported to be an alternative approach to lift the valley degeneracy.^[^ ^107^, ^108^ ^]^ After breaking time‐reversal symmetry, the magnetic moment of valley electrons can interact with magnetic fields via Zeeman‐like interaction (known as valley Zeeman effect). In the independent‐particle picture, the Zeeman splitting is a magnetic field‐induced one with opposite signs for two valleys, contributed by the combination of spin, intra‐atomic *d* orbital, and valley orbital magnetic moment (Figure 5d). To express the Zeeman energy quantitatively, Landé *g*‐factor that is defined as dimensionless magnetic moment is widely adopted. For monolayer WSe_2_, polarization‐resolved PL under magnetic fields revealed the valley splitting of ≈0.11 ± 0.01 me V^−1^ and corresponding *g*‐factor up to 2.^[^ ^107^ ^]^ Meanwhile, the valley splitting under magnetic fields also lifted the valley degeneracy and successfully manifested the valley polarization (Figure 5e,f). Up to now, the observed valley Zeeman splitting by several independent groups is in the order of 0.07–0.36 meV^−1^, corresponding to *g*‐factors with magnitudes of 1–6.^[^ ^107^, ^108^, ^109^, ^110^, ^111^, ^112^, ^113^, ^114^, ^115^, ^116^, ^117^ ^]^ Noting that the neutral A excitons ($X_{A}^{0}$), B excitons ($X_{B}^{0}$), and charged (*X* ^±^) excitons for TMDs are selectively investigated for distinct TMDs in these studies, we summarized the valley splitting and corresponding *g*‐factors in **Table** **1** for intuitive view. It is seen that the majority of *g*‐factor for pristine monolayer TMDs is less than 6, implying the inadequate manipulation of magnetic fields. To overcome this drawback, doping was experimentally proven to significantly enhance valley Zeeman effect in monolayer TMDs through the strong electron–electron interactions.^[^ ^118^, ^119^, ^120^, ^121^, ^122^ ^]^ The *g*‐factor in monolayer WSe_2_ by reducing the hole density approaches 12, two times higher than the predicted value by single‐particle model.^[^ ^119^, ^121^ ^]^

**Table 1:** Summary of effective *g*‐factors in monolayer TMDs

Material	$X_{A}^{0}$ *g*‐factor	$X_{B}^{0}$ *g*‐factor	*X* ^±^ *g*‐factor	Ref
WS_2_	−3.94 ± 0.04	−3.99 ± 0.04	—	[112]
	−3.85	—	−5.04	[113]
MoS_2_	−4.0 ± 0.2	−4.65 ± 0.17	—	[112]
	−1.36	—	—	[113]
	−3.83 ± 0.05	−4.06 ± 0.07	—	[114]
	−4.3 to −4.7	−4.1 to −4.4	—	[111]
WSe_2_	−3.7 ± 0.2	—	−4.4 ± 0.2	[116]
	4.37 ± 0.15	—	6.28 ± 0.32	[110]
	−1.9 ± 0.2	—	—	[107]
	≈4	—	≈4	[115]
	—	—	4 ± 0.5	[117]
	—	—	12	[121]
	—	—	11.7	[119]
	—	—	8.5	[122]
MoSe_2_	−3.8 ± 0.2	—	−3.9 ± 0.2	[116]
	−3.8 ± 0.2	—	−3.8 ± 0.2	[109]
	−3.8 to −4.5	—	—	[111]
	—	—	13	[118]

### 4.5 Circular Photogalvanic Effect

**Figure 6:**
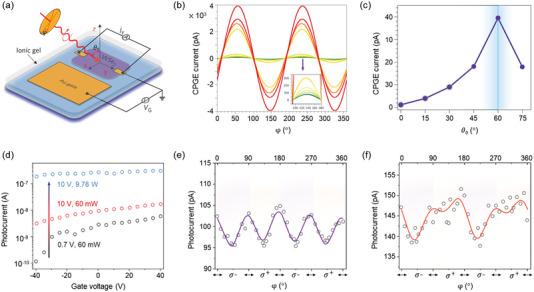
Circular photogalvanic effect in TMDs system. a) Schematic diagram of a WSe_2_ electric‐double‐layer transistor with ionic gel gating. b) Light‐polarization dependent CPGE photocurrent (*j*_CPGE_) in WSe_2_ electric‐double‐layer transistors at external gate voltages (*V*_g_) varied from 0 to 1.1 V. Inserted image shows the *j*_CPGE_ at *V*_g_ < 0.7 V. c) CPGE photocurrent as a function of the incident angle (*θ*_0_). Reproduced with permission.^[^ ^125^ ^]^ Copyright 2014, Springer Nature. d) Photocurrent of monolayer MoS_2_ illuminated by 2.33 eV laser, for *φ* = 0° and *θ*_0_ = 45°. e,f) CPGE photocurrent as a function of light helicity (*φ*) for monolayer MoS_2_ under the illumination of 2.33 eV (off‐resonance) and 1.96 eV (on‐resonance) lasers. The purple and red curves are the fit functions. Reproduced with permission.^[^ ^123^ ^]^ Copyright 2015, Springer Nature.

Different from graphene and BP, the experimental realization of CPGE in TMDs is feasible, especially in their monolayer counterpart with direct bandgap and broken inversion symmetry.^[^ ^123^, ^124^ ^]^ Basically, the CPL absorption of TMDs leads to the angular momenta transformation from photons to electrons; and consequently, the non‐equilibrium spin polarization of electrons forms spin‐coupled valley photocurrent. **Figure** **6**a sketches a WSe_2_ transistor with ionic gel gating.^[^ ^125^ ^]^ In their experiment, the gate potential drove the ionic ions onto the WSe_2_ surface and the newly‐formed perpendicular electric field broke the inversion asymmetry of WSe_2_. Figure 6b,c exhibits the corresponding CPGE amplitude tuned by electric fields and the incident angle of CPLs. Through density functional theory, the authors concluded that the CPGE current was a pure valley‐polarized current or a partially spin‐polarized photocurrent depending on whether spin–orbit coupling was ignored or not. After that, Eginligil et al.^[^ ^123^ ^]^ reported the helicity‐dependent photocurrent in monolayer MoS_2_ under on/off‐resonance photo‐excitations (2.33 eV and 1.96 eV, respectively). Low gate (*V*_g_ = 0.7 V) and illumination (60 mW cm^−2^) were used in their experiments to prevent artificial photocurrent generation (Figure 6d). For off‐resonance excitation (2.33 eV), there was no CPGE photocurrent difference between *σ*+ and *σ*− excitations corresponding to a negligible PC polarization. However, under 1.96 eV excitation, the CPGE photocurrent showed dependence on the angle of photon polarization (Figure 6e,f) and yielded a large polarization of ≈60%.

As an effective means to detect and manipulate the spin and valley degrees of freedom, CPGE was also verified in MoSe_2_^[^ ^126^ ^]^ and WS_2_^[^ ^127^ ^]^ and is gradually becoming a hot topic in the field of spintronics and valleytronics. However, more research efforts are still needed to establish a unified theory to effectively screen the TMDs library with outstanding CPGE performances.

## 5 Heterostructures

Van der Waals (vdWs) heterostructures that artificially stack multiple 2D layers and are coupled together by van der Waals force, attract intense attention from the joint community of chemistry, materials, and physics. In addition to reserving the extraordinary properties of each constituent 2DMs, vdW heterostructures exhibit new fascinating physical phenomena, such as valley polarization, valley Hall, and Zeeman effects and other valley‐contrasting related effects.^[^ ^128^, ^129^, ^130^ ^]^ In addition, the atomic alignment of two 2DMs layer exhibits periodic variations that is defined as moiré superlattice. Accompanied with in‐plane superlattice, the as‐generated moiré interlayer excitons with specific optical selective rules become a versatile platform for exploring exciton‐ and valley‐related physics.^[^ ^131^ ^]^ More importantly, the crystallographic alignment, external fields, and substrate engineering were also demonstrated to ignite and tune these emerging phenomena.^[^ ^132^ ^]^

### 5.1 Interlayer Excitons

Among tremendous vdWs heterostructures, TMDs‐based heterostructures have taken the centerstage for studying interlayer exciton, especially those with type‐II band alignment.^[^ ^133^, ^134^, ^135^, ^136^, ^137^ ^]^ In Mo*X*_2_/W*X*_2_ heterostructure (where *X* = Se, S), for instance, the conduction band minimum (CBM) of Mo*X*_2_ is usually lower with respect to that of W*X*_2_. As a result, interlayer charge transfer occurs spontaneously driven by the difference of Fermi levels. After optical excitations, electrons accumulate in the CB of the Mo*X*_2_ layer with holes in the VB of W*X*_2_ layer. Owing to strong Coulomb interactions, “hole–electron” pairs form by bonding the electrons and holes from adjacent layers, also renamed as interlayer excitons. Due to electron and hole localized in different layers, the overlapping of electron and hole wavefunctions in interlayer excitons is reduced and prolonged lifetimes of interlayer excitons can be expected (ns to µs).^[^ ^138^, ^139^, ^140^ ^]^ In a parallel report, Coulomb exchange interactions are greatly suppressed because the holes and electrons reside in separate W*X*_2_ and Mo*X*_2_ layer, respectively. Profiting from the physical separation, the lifetime of valley excitons approaches up to 40 ns and breaks the picosecond lifetime limitation of valley excitons in monolayer TMDs.^[^ ^141^ ^]^ Certainly, single particle such as resident hole in p‐type TMD monolayers provides alternative approach for achieving longer valley lifetime by eliminating electron–hole exchange interaction and decoherence process. In addition, the huge spin–orbit splitting and spin‐valley locking constrain spin flipping for any valley scattering, elevating the valley polarization lifetime in the range of hundred nanoseconds to several microseconds.^[^ ^142^, ^143^ ^]^ Next, we will focus on the fascinating valley polarization,^[^ ^141^ ^]^ valley Hall,^[^ ^144^ ^]^ and Zeeman effects^[^ ^129^ ^]^ in LMDs‐based heterostructures.

#### 5.1.1 Valley Polarization

**Figure 7:**
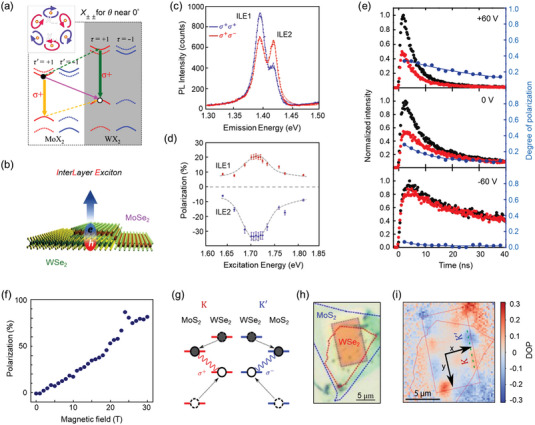
Valley polarization and valley Hall effect of interlayer excitons. a) Predicted valley‐dependent optical selection rules for interlayer exciton *X*_++_ for *θ* = 0°. Solid (dashed) arrows indicate the dipole transition (interlayer hopping). The valley indices (*τ*, *τ*′) correspond to (+, +) or (−, −). The inset shows the elliptical polarization of emissions in six light cones. Reproduced with permission.^[^ ^145^ ^]^ Copyright 2015, American Physical Society. b) Schematic representation of the MoSe_2_/WSe_2_ heterostructure. c) PL measurements for MoSe_2_/WSe_2_ heterostructure under CPLs at 1.71 eV (725 nm) with the same (blue) and opposite (red) helicity at 5K. d) Circular polarization as a function of excitation energy. Reproduced with permission.^[^ ^148^ ^]^ Copyright 2018, American Chemical Society. e) Time‐resolved interlayer exciton PL under *σ*+ pulsed laser excitation at *V*_g_ = −60, 0, and +60 V. Co‐polarized and cross‐polarized PL are shown in black and red curves, respectively. The blue curve shows the valley polarization dynamics. Reproduced with permission.^[^ ^141^ ^]^ Copyright 2016, AAAS. f) Valley polarization of interlayer exciton as a function of out‐of‐plane magnetic field. Reproduced with permission.^[^ ^129^ ^]^ Copyright 2017, Springer Nature. g) Interlayer exciton in different valleys. Electrons (grey dots) hop to the conduction band of MoS_2_ and Holes (empty dots) hop to the valence band of WSe_2_. h) Optical image of heterostructure device. The blue (red) dashed line indicates monolayer MoS_2_ (WSe_2_). A rectangular silicon suspended slab was fabricated in the middle of the substrate. i) Experimental demonstration of valley Hall effect in WSe_2_/MoSe_2_ heterostructure at room temperature. The green linecut represents the distribution of the degree of polarization. Reproduced with permission.^[^ ^150^ ^]^ Copyright 2019, American Chemical Society.

**Figure** **7**a shows the valley polarization (VP) in twisted Mo*X*_2_/W*X*_2_ heterostructure (twist angle *θ* = 0° or 60°). In accordance to polarization selection rules, the interlayer excitons in the light cones were simulated to be able to couple with light.^[^ ^145^ ^]^ Experimentally, the interlayer excitons and valley polarization were realized via the circularly polarized PL measurements in MoSe_2_/WSe_2_ heterostructures.^[^ ^146^, ^147^ ^]^ Up to date, the best VP value of interlayer excitons was verified to approach ≈30% in MoSe_2_/WSe_2_ heterostructure without any electrical‐ or magnetic‐field modulations.^[^ ^141^ ^]^ In the same heterostructure, interlayer excitons were found to transit between the highest valence state and two lowest spin–orbit‐split conduction states; and consequently, the emission peaks of interlayer excitons showed opposite polarizations (VP value at 20% and −35%, Figure 7b–d) under different CPLs (*σ*+ and *σ*−), respectively.^[^ ^148^, ^149^ ^]^ When conversely stacking WSe_2_ monolayer onto MoSe_2_ monolayer, Hsu et al. reported negative circular polarization of interlayer exciton emission and ascribed the polarization originating from the quantum interference induced by interlayer atomic registry.^[^ ^146^ ^]^

For certain TMDs‐based heterostructure toward valleytronic applications, efficient methods to tune the magnitude of valley polarization are highly demanded. In MoSe_2_/WSe_2_ heterostructures, Rivera et al.^[^ ^141^ ^]^ pioneerly demonstrated the controllable VP by a gate voltage. From the time‐resolved PL data, the VP value from interlayer exciton reached the maximum (≈40%, valley lifetime ≈40 ns) at *V*_g_ = +60 V but was extremely suppressed at *V*_g_ = −60 V (Figure 7e). The authors explained that the outstanding VP value came from the weaker Coulomb exchange interactions and suppressed intervalley scattering induced by the separation of electron–hole layer. However, a clear microscopic mechanism for the gate‐dependent valley polarization dynamics of interlayer excitons was left unknown. In another report that also focused on MoSe_2_/WSe_2_ heterostructure, the emission polarization of interlayer excitons was even switched to be negative/positive by gate voltages.^[^ ^149^ ^]^ As a substitute of electrical fields, magnetic field was also employed to manipulate valley polarization of TMD heterostructures. By exposing zero‐twisted MoSe_2_/WSe_2_ heterostructure under intense magnetic fields (28 T), Nagler et al.^[^ ^129^ ^]^ observed the valley polarization up to ≈80% and the lifetime of interlayer excitons approaching 70 ns (Figure 7f), the best valley polarization and lifetime values to our limited knowledge.

Apart from comparable exciton lifetime and valley depolarization time to their monolayer counterparts, the reported valley polarization of interlayer excitons in TMDs‐based heterostructure is somehow unsatisfying. The limiting factors may lie in sample inhomogeneity, indirect band‐gap transitions,^[^ ^148^, ^149^ ^]^ and slow charge separation.^[^ ^141^ ^]^ Meanwhile, it is also worthy to mention that the valley selection rules of interlayer excitons here still remain debated. To solve these obstacles, more research efforts are demanded to reveal the underlying mechanisms and push the magnitude of VP close to the theoretical value.

#### 5.1.2 Valley Hall Effect

In TMDs‐based heterostructures, the weak interlayer‐band hybridization in the K and K′ valleys enables interlayer excitons possessing valley‐dependent Berry curvatures, giving rise to an exciton valley Hall effect. Under CPL, the interlayer excitons generate an anomalous transverse velocity perpendicular to the carrier flow direction, resulting in the spatial splitting of interlayer exciton populations on the basis of valley polarization.

In light of valley‐contrasting optical selection rule, excitons in different valleys can be detected with exclusive optical helicity. Figure 7g shows the room‐temperature exciton VHE in MoS_2_/WSe_2_ heterostructures.^[^ ^150^ ^]^ In their work, a suspended slab geometry was used to create strains at the edges of the slab and the induced potential gradient enabled interlayer excitons to acquire valley‐dependent transverse velocities (Figure 7h). Resolved by polarization‐dependent PL spectroscopy, interlayer excitons were observed to separate along the transverse directions (Figure 7i). In the same MoS_2_/WSe_2_ heterostructure, Jiang et al.^[^ ^144^ ^]^ recently observed the room‐temperature VHE via gate‐tunable Hall voltage measurements. More importantly, the authors precisely manifested the magnitude and polarity of VHE by gate voltages resulting from the opposite contributions from carriers residing in different layers and the electrically induced valley‐dependent band‐shift.

The observation of VHE in monolayer TMDs (usually based on intralayer excitons) inevitably requires either cryogenic temperature^[^ ^93^, ^95^ ^]^ or plasmonic structures^[^ ^151^ ^]^ because of the shorter valley lifetimes and enhanced valley depolarization at elevated temperature. In contrast, profiting from extended valley lifetimes by electron–hole separation in different layers, interlayer excitons from TMD‐based heterostructure were more favorable to preserve VHE at higher temperature and even room temperature, creating many possibilities for implementing the valley as information carriers in next‐generation electronics.

#### 5.1.3 Valley Zeeman Effect

**Figure 8:**
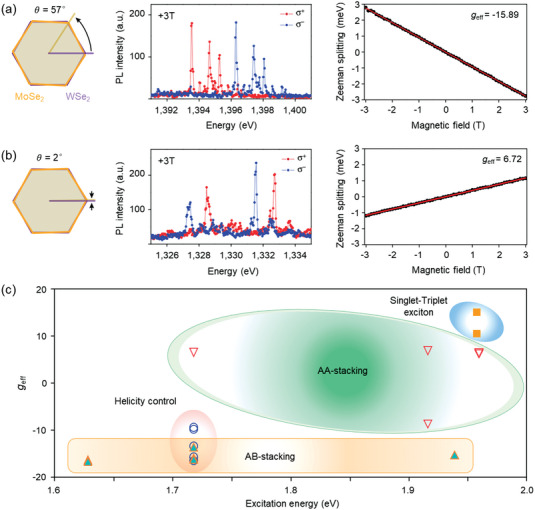
Valley Zeeman effect in MoSe_2_/WSe_2_ heterostructure. Magnetic‐field‐dependent PL from interlayer excitons in MoSe_2_/WSe_2_ heterostructure with twist angles of a) 57° and b) 2°. Left: Schematic diagrams of the heterostructure with twist angles. Middle: helicity‐resolved PL under linearly polarized excitation at 3 T. Right: Zeeman splitting as a function of external magnetic fields. The derived effective *g*‐factors from Zeeman splitting are −15.89 ± 0.02 and 6.72 ± 0.02 for samples with twist angles of 57° and 2°, respectively. Reproduced with permission.^[^ ^154^ ^]^ Copyright 2019, Springer Nature. c) Reported effective *g*‐factors of interlayer excitons in the MoSe_2_/WSe_2_ heterostructures.

The Valley Zeeman splitting of interlayer excitons was found to be more intense than that of intralayer excitons reflected by measuring the effective *g*‐factors.^[^ ^152^ ^]^ According to the literature, the *g*‐factor values of the interlayer excitons in TMDs‐based heterostructures heavily depend on the stacking sequence and configuration, spin properties of heterostructure (**Figure** **8**), external magnetic field, as well as the helicity of excitation light.^[^ ^149^, ^153^, ^154^, ^155^ ^]^

Figure 8a,b shows the stacking‐dependent *g*‐factors of interlayer excitons in MoSe_2_/WSe_2_ heterostructure and the corresponding *g*‐factors were ≈ −15.9 and ≈6.7 for the AB‐stacking and AA‐stacking, respectively, under the same testing conditions.^[^ ^154^ ^]^ The larger effective *g*‐factors with opposite sign in AB‐stacking with respect to AA‐stacking ones may originate from distinct conduction‐valence valley pairings. In particular, the valley index pairs for the conduction and valence bands were ((+, +) or (−, −)) and ((+, −) or (−, +)) for AA‐stacking and AB‐stacking, respectively. Hence, the valley contribution was additive in the AB‐stacked one and produced a larger effective interlayer exciton *g*‐factor.^[^ ^129^, ^155^ ^]^ Figure 8c shows the spin‐dependent *g*‐factors comparison of interlayer excitons in TMDs‐based heterostructure.^[^ ^156^, ^157^ ^]^

It is also recognized that triplet interlayer excitons correspond to spin‐flip transitions; and consequently, provide an extra spin contribution to the *g*‐factor. Experimentally, Wang et al. demonstrated that spin‐triplet interlayer excitons in AB stacked MoSe_2_/WSe_2_ showed giant valley‐Zeeman splitting with *g*‐factor of ≈15.2, larger than the *g*‐factor (≈10.7) of spin‐singlet.^[^ ^157^ ^]^ Last but not the least, the helicity of excitation lights and the direction of external magnetic fields were synergized together to improve the *g*‐factors up to ≈ −16 in ordinary TMDs‐based heterostructure.^[^ ^158^, ^159^ ^]^

### 5.2 Moiré Excitons

**Figure 9:**
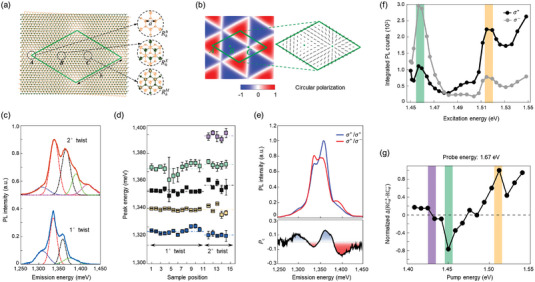
Fundamental properties of moiré interlayer exciton. a) Moiré pattern in an R‐type Mo*X*_2_/W*X*_2_ heterostructure. Green diamond outlines the moiré supercell in real space. Insets are three highlighted regions, where local atomic registries resemble lattice‐matched bilayers of different R‐type stacking. b) Optical selection rule for interlayer exciton emission at three highlighted regions. Reproduced with permission.^[^ ^163^ ^]^ Copyright 2017, AAAS. c) Representative PL for MoSe_2_/WSe_2_ heterostructures with twist angles of 1° (bottom) and 2° (top). d) The center energy of each moiré interlayer exciton at different spatial positions. e) Circularly polarized PL of 1° twisted sample under *σ*+ excitation light. Bottom panel is the degree of circular polarization as a function of the emission wavelength. Reproduced with permission.^[^ ^131^ ^]^ Copyright 2019, Springer Nature. f) Helicity‐resolved PL excitation to probe interlayer moiré exciton states in the WSe_2_/WS_2_ heterostructure. The green (1.46 eV) and yellow (1.51 eV) shaded area represent two new interlayer moiré exciton states. g) Pump‐induced circular dichroic signal with probe energy fixed at 1.67 eV and pump energy swept from 1.38 to 1.54  eV. Reproduced with permission.^[^ ^164^ ^]^ Copyright 2019, Springer Nature.

Assembling TMDs heterostructures with small lattice constant mismatch (≈0.1%) and/or relative orientation angle (excluding angle ≤1°^[^ ^160^ ^]^) creates moiré patterns or superlattice in which the configurations of interlayer atomic changes periodically. The corresponding moiré periodicity *a*_M_ is given by

(2) $$a_{M}\approx a_{0}/\sqrt{\theta^{2}+\delta^{2}}$$

where *δ* is the lattice constant mismatch defined as $|a_{0}-a_{0}^{\prime }|/a_{0}$, *a*_0_ and$\;a_{0}^{\prime }$ are the lattice constants of the two layers, and *θ* is the relative twist angle.^[^ ^161^ ^]^ In the periodic superlattice with varying interlayer atomic registries, three high‐symmetry points (*A*, *B*, and *C*) are formed in the moiré supercell with the threefold rotational symmetry$\;{\hat{C}}_{3}$.^[^ ^162^ ^]^ The corresponding interlayer atomic configurations at their rotation center are$\;R_{h}^{h}$,$\;R_{h}^{X}$, and $R_{h}^{M}$ registries (**Figure** **9**a), where $R_{h}^{\mu }$ denotes an R‐type stacking with the *µ* sites of electron layer on the hexagon center (h) of hole layer (*X*: chalcogen; *M*: metal). These three high‐symmetry points are local energy extrema and localize interlayer excitons in the Moiré supercell. According to theoretical calculation, the localized interlayer exciton states at three high‐symmetry points had distinct optical selection rules.^[^ ^161^, ^163^ ^]^ In particular, the interlayer excitons located at the *A* (*B*) registries (Figure 9b) were coupled only to *σ*+ (*σ*−) circularly polarized light but the coupling was forbidden for the excitons at *C* point. For other regions in the moiré supercell, the emissions were elliptically polarized. The interlayer excitons with the same spin‐valley configuration might also couple with certain polarized light with opposite helicity, suggesting the breaking of the optical selection rules of exciton and the unlocking of valley index.

To probe the influence of moiré superlattice upon interlayer excitons, Tran et al.^[^ ^131^ ^]^ fabricated MoSe_2_/WSe_2_ heterostructures with a small twist angle and observed multiple interlayer exciton resonances via micro‐photoluminescence measurements. The resonance energies at different spatial positions showed nearly constant peak spacing of 22 ± 2 meV (Figure 9c–e), probably originating from the ground‐ and excited‐state interlayer excitons laterally confined within a moiré potential of ≈100 meV. Soon after, in WSe_2_/WS_2_ heterostructures with near‐zero twist angle, background‐free techniques including photoluminescence excitation and resonant pump–probe spectroscopy were used to probe the multiple interlayer exciton states.^[^ ^164^ ^]^ Figure 9g shows two opposite pump–probe signals at 1.46 and 1.51 eV, corresponding to two interlayer exciton states showing different helicity (Figure 9f). Moiré interlayer exciton states in the K valley can couple to *σ*− and *σ*+ lights (at 1.46 and 1.51 eV), exhibiting opposite optical selection rules due to their different moiré quasi‐angular momentum arising from the local interlayer atomic registry.

Combining the property of multiple moiré states with distinct optical selection rules, moiré excitons in TMDs‐based heterostructures become a promising platform for exploring valley physics. However, the assembly of TMDs‐based heterostructure at current stage requires much manpower and the avoidable non‐homogeneities from the fabrication process may broaden the linewidths of interlayer excitons. To avoid these problems, mature technologies for preparing heterostructures with specific angles are urgently required.

### 5.3 Magnetic Proximity Effect

**Figure 10:**
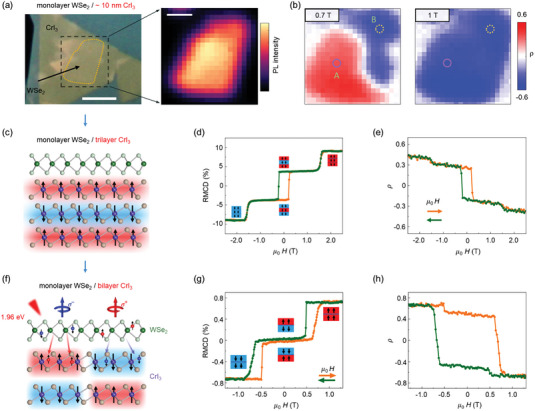
Magnetic proximity effect in WSe_2_/CrI_3_ heterostructure. a) Left: optical image of monolayer WSe_2_/multilayer CrI_3_ (≈10 nm) heterostructure. Laser‐scanning area is marked by the dashed box and monolayer WSe_2_ is defined by dotted yellow curve. Right: spatial map of WSe_2_ PL intensity in the dashed box region. b) Spatial maps of the polarization (*ρ*) under 1 T (right) and 0.7 T (left) applied magnetic fields. Reproduced with permission.^[^ ^132^ ^]^ Copyright 2018. American Chemical Society. c) Schematic of monolayer WSe_2_/trilayer CrI_3_ heterostructure. d) Reflective magneto‐circular dichroism (RMCD) as a function of applied magnetic field. Orange and green curves indicate magnetic field sweeping up (increase) and down (decrease), respectively. e) Degree of circular polarization (DP) as a function of applied magnetic field. f) Schematic of monolayer WSe_2_/bilayer CrI_3_ heterostructure. g) Reflective magneto‐circular dichroism as a function of applied magnetic field, exhibiting typical features of a layered antiferromagnetic bilayer CrI_3_. h) DP as a function of magnetic field. Reproduced with permission.^[^ ^166^ ^]^ Copyright 2020, Springer Nature.

The magnetic proximity effect usually refers to the introduction of magnetic moment and magnetic order in non‐magnetic material by adjacent magnetic material because of large magnetic exchange field.^[^ ^165^, ^166^ ^]^ In heterostructures consisting of TMDs monolayers on ferromagnetic insulators, the magnetic exchange fields at the interface break the time‐reversal symmetry of monolayer TMDs and further manipulate the valley polarization and valley Zeeman splitting.^[^ ^167^, ^168^, ^169^, ^170^ ^]^ For example, magnetic thin CrI_3_ crystal was first attempted (**Figure** **10**) to provide interfacial magnetic exchange field. Figure 10a exhibits the generation of magnetic exchange field (≈13 T) in monolayer WSe_2_/multilayer CrI_3_ (≈10 nm) heterostructure. Based on this magnetic proximity effect, the magnitude and sign of valley polarization (Figure 10b) could be tuned by external magnetic field in an elegant way.^[^ ^132^ ^]^ Due to the dependent ferromagnetism/antiferromagnetism, bilayer and trilayer CrI_3_ were then employed to probe the magnetic proximity effect in WSe_2_/CrI_3_ heterostructures. In monolayer WSe_2_/trilayer CrI_3_ heterostructure (Figure 10c), the degree of circular polarization (*ρ*) signal of WSe_2_ switched between positive and negative (Figure 10d,e) when the topmost CrI_3_ layer flipped the magnetization.^[^ ^166^ ^]^ Similar observation was also corroborated in monolayer WSe_2_/bilayer CrI_3_ heterostructure (Figure 10f–h), implying that spin‐dependent charge transfer occurs at the WSe_2_/CrI_3_ interface and is mainly dominated by the topmost CrI_3_ layer.

The CrI_3_ validation as heterostructure building blocks for probing magnetic proximity effects stimulates intense explorations of fascinating magnetic 2D crystals. In this circumstances, Zhao et al.^[^ ^168^ ^]^ selected ferromagnetic EuS for supporting TMDs and reported a giant valley splitting because of the interfacial magnetic exchange fields at monolayer WSe_2_/EuS interface. By utilizing magneto‐reflectance measurements, the effective magnetic exchange field was estimated to be ≈12 T and the valley splitting of WSe_2_ reached as high as 2.5 meV at 1 T. Later, the researcher from the same group obtained the energy splitting of 16 meV T^−1^ in WS_2_/EuS heterostructure (nearly seven times larger than that of WSe_2_/EuS) and the corresponding *g*‐factor was estimated to be ≈ −320. The authors ascribed the valley splitting difference between WSe_2_/EuS and WS_2_/EuS to their distinct band alignment.^[^ ^130^ ^]^ Recently, 2D ferromagnetic Cr_2_Ge_2_Te_6_ semiconductor was employed to construct few‐layer Cr_2_Ge_2_Te_6_/MoSe_2_ heterostructures to study magnetic proximity effect. Owing to interfacial magnetic exchange field, the valley splitting rate was determined to be ≈0.6 meV T^−1^. It is also worthy to note that the valley polarization of monolayer MoSe_2_ is gate‐tunable, creating more rooms for exploring field‐controlled magneto‐optoelectronic devices.^[^ ^171^ ^]^ Remarkably, for TMDs on magnetic substrates (such as CrI_3_), their spatial separations and band alignments, magnetic proximity effect is also governed by the twist angle. In their reports, the valley splitting of MoSe_2_/CrI_3_ and WSe_2_/CrI_3_ heterostructures was enhanced by three‐ or fourfold during increasing the twist angle from 0° to 30°.^[^ ^169^ ^]^ In the absence of magnetic fields, magnetic proximity effect facilitates the valley polarization and Zeeman splitting phenomena in TMDs‐based heterostructure toward low‐power information processing.

## 6 Perovskites

Metal halide perovskites, structural formula of *ABX*_3_ (where *A* represents a monovalent cation, *B* indicates a metallic cation, and *X* denotes a halogen) exhibit outstanding photoelectric properties and become one superstar material in solar cells,^[^ ^172^ ^]^ light‐emitting diodes,^[^ ^173^ ^]^ photodetectors,^[^ ^174^ ^]^ and lasers.^[^ ^175^ ^]^ More importantly, the introduction of chiral ligands into perovskites (namely chiral perovskites) enables themselves fascinating chiral optoelectronic and chiro‐spintronic properties.^[^ ^176^, ^177^ ^]^ Moreover, profiting from the spin–orbit coupling of heavy atoms, many perovskites exhibit outstanding spin‐dependent photophysics.^[^ ^178^, ^179^ ^]^ As a result, circular dichroism,^[^ ^176^ ^]^ CPL sources and detection,^[^ ^177^, ^180^ ^]^ circular photogalvanic effect^[^ ^178^ ^]^ and optical Stark effect^[^ ^181^ ^]^ were successionally reported in distinct perovskites. In this section, the underlying mechanism, figure of merit, and potential optoelectronic applications of perovskites in light of above four phenomena will be systematically overviewed.

### 6.1 Circular Dichroism

The chirality degree of chiral materials is represented by the anisotropy factor (*g*_abs_)^[^ ^182^ ^]^ calculated based on the formula:

(3) $$g_{\mathrm{abs}}=\frac{A_{L}-A_{R}}{\left(A_{L}+A_{R}\right)/2}$$

where *A*_L_ and *A*_R_ are the absorption intensity of the LCP and RCP lights. For chiral perovskites, the crystal orientations, film thickness, and the chemical composition could produce variations of CD signals.

**Figure 11:**
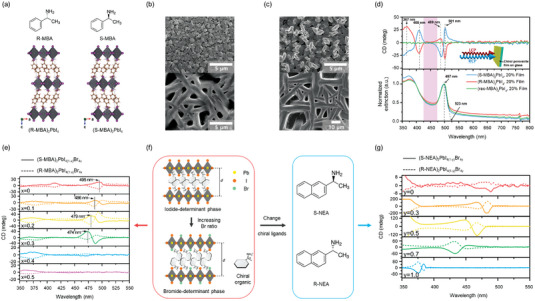
Circular dichroism phenomenon in chiral perovskites. a) Top panel: Molecular structures of S‐MBA and R‐MBA. Bottom panel: Crystalline structures of (S‐MBA)_2_PbI_4_ and (R‐MBA)_2_PbI_4_. b,c) SEM images of different (R‐MBA)_2_PbI_4_ films made from the solutions with concentrations ranging from 20 wt% (top left), 50 wt% (bottom left), 40 wt% (top right), and 66 wt% (bottom right). d) Transmission CD (top panel) and normalized extinction spectra (bottom panel) of (S‐MBA)_2_PbI_4_, (R‐MBA)_2_PbI_4_, and (rac‐MBA)_2_PbI_4_ films. Reproduced with permission.^[^ ^176^ ^]^ Copyright 2017, Royal Society of Chemistry. e) CD spectra of (S‐MBA)_2_PbI_4(1−_ *_x_*_)_Br_4_ *_x_* and (R‐MBA)_2_PbI_4(1−_ *_x_*_)_Br_4_ *_x_* samples (*x* = 0, 0.1, 0.2, 0.3, 0.4, and 0.5). f) Right panel: Iodide/Bromide‐determinant phase of chiral perovskites. Left panel: Molecular structure of S‐ and R‐NEA ligands. g) CD spectra of (S‐NEA)_2_PbI_4(1−_ *_y_*_)_Br_4y_ and (R‐NEA)_2_PbI_4(1−_ *_y_*_)_Br_4_ *_y_* (*y* = 0, 0.3, 0.5, 0.7, and 1.0) samples. Reproduced with permission.^[^ ^183^ ^]^ Copyright 2020, American Chemical Society.

**Figure** **11**a shows the chiral structures of (S‐MBA)_2_PbI_4_ and (R‐MBA)_2_PbI_4_ by incorporating the S‐and R‐methylbenzylamine (S‐MBA and R‐MBA) into layered lead‐iodide framework. After optimizing the crystallization (Figure 11b,c), a desirable *g*_abs_ of 6 × 10^−3^ was reported.^[^ ^176^ ^]^ The chiral CD signals from the same locations exhibited opposite signs (top panel in Figure 11d) and were significantly different from those of the S‐MBA and R‐MBA which are located near 260 nm, evidencing the chirality transfer. Moreover, in the chiral perovskite films, CD peaks appeared before excitonic peaks (497 to 523 nm, bottom panel in Figure 11d) and crossed the zero line at the extinction band edge (497 nm) with sign changing; this arisen phenomenon is due to the splitting of electronic energy levels of perovskite induced by chiral ligands. To probe the influence of crystal orientations and morphologies on chirality, Figure 11b,c shows the controlled crystal orientations and morphologies of perovskite films by adjusting the concentration of precursor solutions. Consequently, the corresponding transmission CD spectra were differentiated because of the orientation‐dependent excitonic transitions.^[^ ^176^ ^]^ Figure 11e shows the tunable CD peaks energy (from 495 to 474 nm) of (S‐ or R‐MBA)_2_PbI_4(1−_ *_x_*_)_Br_4_ *_x_* by controlling the ratio of bromide and iodide anions.^[^ ^183^ ^]^ Similarly, the substitution of S‐ and R‐MBA with larger spacer group (S‐ and R‐NEA) enlarges the band gap of chiral perovskites and then blue‐shifts the CD signals to ≈375 nm (Figure 11g).

### 6.2 Emission and Detection of Circularly Polarized Light

**Figure 12:**
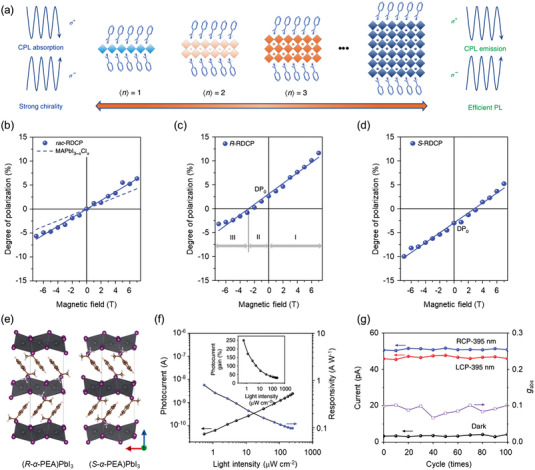
The emission and detections of circularly polarized light in chiral perovskites. a) Schematic diagram of reduced‐dimensional chiral perovskites. b–d) Degree of PL polarization for *rac*‐RDCP (left), R‐RDCP (middle), and S‐RDCP (right) as a function of magnetic fields. Reproduced with permission.^[^ ^177^ ^]^ Copyright 2018, Springer Nature. e) The crystal structure of (*R*‐ and *S*‐*α*‐PEA)PbI_3_ crystals. The gray, rose red, golden yellow, light blue, and fresh pink dots indicate Pb, I, C, N, and H atoms, respectively. f) Photocurrent and responsivity as a function of light intensity (at *V*_bias_ = 20 V under unpolarized 395 nm light). The inset shows light‐intensity‐dependent photocurrent gain. g) The current and *g*_res_ under dark and light illumination (LCP/RCP light: 395 nm) as well as at increased folding cycles. Reproduced with permission.^[^ ^180^ ^]^ Copyright 2019, Springer Nature.

Left‐ and right‐ circularly polarized lights behave as two independent information transmission channels^[^ ^184^ ^]^ and exhibit great promise in bioresponsive imaging,^[^ ^185^ ^]^ information storage,^[^ ^186^ ^]^ and encrypted transmission.^[^ ^187^ ^]^ To emit and detect high‐quality CPL, chiral perovskites materials are reported to directly emit CPL without the premise for additional optics.^[^ ^188^, ^189^, ^190^ ^]^ For instance, quasi‐2D perovskites (**Figure** **12**a) exhibit both CD and spin‐polarized photoluminescence in the absence of external magnetic field.^[^ ^177^ ^]^ To be more specific, the reduced‐dimensional chiral perovskites (RDCPs) could be tuned by manipulating the average number of inorganic layers (⟨n⟩) and the degree of polarization (Figure 12b–d) of chiral (R‐ and S‐RDCP) and achiral RDCPs (*rac*‐RDCP) were measured as a function of external magnetic fields. The degree of polarization was zero for *rac*‐RDCP but exhibited 3% for both R‐and S‐RDCP at 0 T. Here, chiral perovskites were considered as a two‐level system that the emission only originated from one excited state decaying to ground state. The spin‐polarized emission rates of two spin‐allowed transitions become different because of the asymmetric absorption rates of up/down spins accompanied with nonzero degree of polarization for R‐RDCP (or S‐RDCP) at zero magnetic field. It was noteworthy that the degree of polarization in quasi‐2D perovskites was small, and was negligible in pure chiral 2D perovskites. To this end, pure chiral (R‐/S‐MBA)_2_PbI_4_ (MBA = C_6_H_5_C_2_H_4_NH_3_) 2D perovskites were synthesized by Ma et al.^[^ ^188^ ^]^ and exhibited an average degree (≈10%) of circularly polarized PL with a maximum value of 17.6% in (S‐MBA)_2_PbI_4_ at 77 K. Soon after, at room temperature, chiral (*S*)‐*α*‐(PEA)_2_PbI_4_ and (*R*)‐*α*‐(PEA)_2_PbI_4_ were reported to show striking CPL emissions with the average degree of polarization of ≈11.4% and 13.7%, respectively, presumably resulting from the high crystalline.^[^ ^190^ ^]^

In the aspect of CPL detection, chiral perovskites with robust circular dichroism have been attempted as CPL photodetectors to directly differentiate LCP and RCP.^[^ ^180^, ^188^, ^191^, ^192^ ^]^ For instance, chiral perovskites (*R*‐ and *S*‐*α*‐PEA)PbI_3,_ through incorporating the chiral molecule *α*‐phenylethylamine (*α*‐PEA) into organic–inorganic hybrid perovskites (Figure 12e) showed a high responsivity of 797 mA W^−1^ (Figure 12f) and an anisotropy factor (*g*_abs_) of ≈0.1 (Figure 12g).^[^ ^180^ ^]^ However, there exists a trade‐off between the different CPL absorption and photocurrent responsivity in chiral perovskites, probably limited by the development of suitable chiral materials, film crystallization, and even device architecture. Therefore, the search for perovskite‐based CPL photodetectors with both outstanding *g*_abs_ and responsivity is promising in the near future.

### 6.3 Circular Photogalvanic Effect

**Figure 13:**
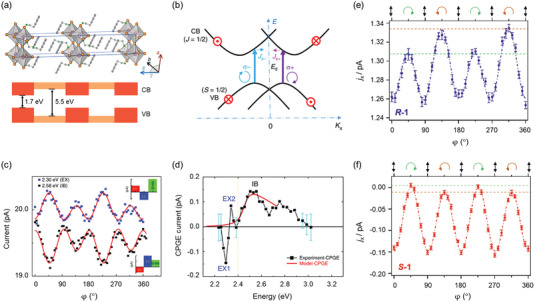
Circular photogalvanic effect in perovskites. a) Schematic of 2D‐PEPI perovskite. b) Illustration of Rashba splitting in the continuum bands and interband transitions under CPL. c) The plots of CPGE current and light helicity, using an excitation with 2.30 eV (blue squares) for exciton (EX) generation, and 2.64 eV (black squares) for interband excitation, respectively. d) The CPGE (*C1*) action spectrum of 2D‐PEPI crystal (black squares). The red line through the data points is a fit. The spectrum is divided by a vertical dashed line into two spectral ranges, exciton and interband. The two exciton species are labeled as exciton 1 (EX1) and exciton 2 (EX2). Reproduced with permission.^[^ ^193^ ^]^ Copyright 2020, Springer Nature. e,f) The total photocurrent (*j_x_*) versus light polarization (*φ*) curves. The blue *(R*‐1) and red (*S*‐1) chain‐dotted lines correspond (*R*‐BrPEA)_2_PbI_4_ and (*S*‐BrPEA)_2_PbI_4_ samples, respectively. Reproduced with permission.^[^ ^178^ ^]^ Copyright 2021, Wiley‐VCH GmbH.

The heavy atoms in perovskite materials usually lead to the spin splitting of electronic band because of spin–orbit coupling (SOC), accompanied by the circular photogalvanic effect that helps to verify the existence of Rashba effect for quantum well and distorted structures. 2D‐phenethylammonium lead iodide (2D‐PEPI) possesses natural multiple quantum wells (**Figure** **13**a) that the inorganic layers ([PbI_6_]^4−^) and the organic cation chains (C_6_H_5_C_2_H_4_NH_3_^+^) served as potential “wells” and “walls”, respectively.^[^ ^193^ ^]^ At the interfaces/surfaces with broken inversion symmetry, a giant Rashba splitting occurs in electronic bands. For two Rashba branches, the spin polarizations become non‐equilibrium because of different absorption between RCP (*σ*+) and LCP (*σ*−) lights, producing the CPGE photocurrent (*J_x+_* or *J_x_*_−_, Figure 13b). As can be seen in Figure 13c, the induced photocurrents showed a dependence on the CPL helicity based on the phenomenological CPGE expression. Meanwhile, two responses had opposite signs for resonant excitation at the exciton and interband transitions, and the corresponding CPGE variations under two excitations (Figure 13d) were due to photo‐generated excitons and free carriers created via interband transition. Second, thermally assisted structural distortion was also found to activate CPGE current.^[^ ^194^ ^]^ In 2D Dion–Jacobson perovskite (AMP)PbI_4_ crystals, CPGE was proven to vary periodically with the helicity of incident light. The strong temperature‐dependent PL helicity also confirmed that thermally assisted structural distortion was the main reason for CPGE current due to the persistent valley polarization at high temperatures. Obviously, CPGE was also realized in chiral perovskites (*R/S*‐BrPEA)_2_PbI_4_ (Figure 13e,f)^[^ ^178^ ^]^ and (*R*/*S*‐MBA)_2_PbI_4_^[^ ^179^ ^]^ that lack centro‐symmetry. More importantly, these responses from two chiral counterparts showed opposite signs because of inverted spins in radial spin textures benefiting from the intrinsic chirality of perovskite crystals. These works demonstrated a rational strategy to design novel opto‐spintronic devices by chirality control.

### 6.4 Optical Stark Effect

**Figure 14:**
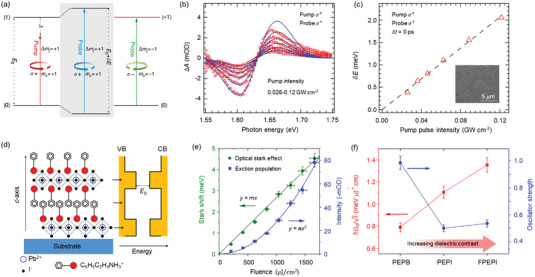
Optical Stark effect in perovskites. a) Schematic of the exciton energy shift induced by OSE. b) The TA variation recorded at time zero under different pump intensities (red circles). c) Energy shift of the exciton transition as a function of pump pulse intensities. Inset is the SEM image of the top‐view of the lead iodide perovskite sample. Reproduced with permission.^[^ ^181^ ^]^ Copyright 2016, Springer Nature. d) Schematic PEPI structure. CB, conduction band; VB, valence band. e) Optical Stark shift and exciton population as a function of pump fluency. f) Rabi energy (which parameterized the light–matter coupling strength) due to OSE and oscillator strength for PEPB, PEPI, and FPEPI perovskite materials. Reproduced with permission.^[^ ^195^ ^]^ Copyright 2016, AAAS.

Profiting from the discrete energy levels and strong bound edge transition with large oscillator strength, 2D layered lead halide perovskites and perovskite quantum dots provide a fascinating platform for investigating optical Stark effect.^[^ ^195^, ^196^, ^197^ ^]^ **Figure** **14**a exhibits the OSE‐induced energy shift of exciton transition of lead iodide perovskites by non‐resonant pump photon field. More specifically, the *σ*+ photon coupled only to the *m*_j_ = +1 excitonic state and generated new entangled state that was detected by a probe *σ*+ photon. Meanwhile, the opposite‐spin exciton state (*m*_j_ = −1) remained unperturbed. Furthermore, polarization‐selective OSE was observed in methylammonium lead iodide (MAPbI_3_) perovskite films at room temperature, in accordance to spin‐selective rule.^[^ ^181^ ^]^ In their study, the OSE showed a linear relationship between the shift of exciton transition energy and pump pulse intensity (Figure 14b). When the pump intensity reached 21.9 µJ cm^−2^, an OSE‐induced energy shift ≈ 2 meV was obtained (Figure 14c). Similarly, Giovanni et al.^[^ ^195^ ^]^ demonstrated the ultrafast spin‐selective OSE in solution‐processed perovskite thin films under room temperature by transient absorption spectroscopy. Figure 14d shows the multiple quantum well structure of (C_6_H_5_C_2_H_4_NH_3_)_2_PbI_4_ with alternating organic (C_6_H_5_C_2_H_4_NH_3_^+^) and inorganic ([PbI_6_]^4−^ octahedron) layers. The Stark shift versus pump fluency exhibited a linear dependence (Figure 14e) and approached up to ≈4.5 ± 0.2 meV at the pump fluency ≈1.66 mJ cm^−2^. Notably, by substituting the halide component from bromide to iodide (PEPB to PEPI), the coupling strength of light and perovskites via OSE increased with the enhancement of dielectric contrast (Figure 14f). This phenomenon could be attributed to the enhanced light–matter interaction (OSE and oscillator strength) by the introduction of high‐quality microcavity with strong photon modal confinement. Recently, spin‐selective OSE has also been comprehensively investigated in perovskite quantum dots at room‐temperature.^[^ ^196^ ^]^ In accordance to their reports, the observed OSE shift could approach up to 6.4 meV at a pump intensity of 0.71 GW cm^−2^ for CsPbI_3_ quantum dots (diameter ≈8.3 nm) through the same working principles as mentioned earlier. These demonstrations of manipulating OSE in perovskites at room temperature would constitute a big step for future spintronic applications.

## 7 Quasi‐2D Topological Materials

Topological insulators (TIs) possess metallic topological surface states (TSSs) with Dirac‐like dispersions and insulating bulk states with an energy gap.^[^ ^198^, ^199^, ^200^ ^]^ Different from gapless graphene, the surface states of TIs are the spin‐momentum locking and the spins constitute helical spin‐momentum textures by cycling around a constant‐energy contour of the Dirac‐cone surface state.^[^ ^201^ ^]^ The CPL illumination upon this spin‐momentum locking surface would produce spin polarization and spin current, enabling many exotic phenomena including spin polarization, circular photogalvanic effect, and spin Hall effect. Here, quasi‐2D layered TIs (Bi_2_Se_3_, Sb_2_Te_3_, and Bi_2_Te_2_Se) that strongly interact with circularly polarized lights are mainly focused and overviewed.

### 7.1 Spin Polarization

**Figure 15:**
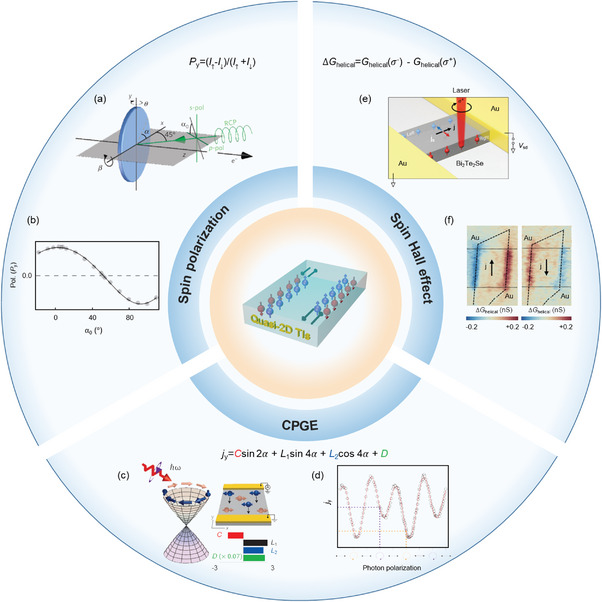
CPL‐related phenomena in quasi‐2D topological insulators. a) Schematic diagram of the experimental setup. Linear polarization of photons can be continuously rotated. b) Photoelectron spin polarization (*P_y_*) as a function of continuously rotating the photon polarization. The black curve is a theoretical fit. Reproduced with permission.^[^ ^204^ ^]^ Copyright 2013, Springer Nature. c,d) Surface photocurrent (*j_y_*) from helical Dirac fermions with light oblique incidence in the *x–z* plane at 15 K. The solid red line (right graph) is fitted following the equation (top panel). Reproduced with permission.^[^ ^210^ ^]^ Copyright 2011, Springer Nature. e) Sketch of the experimental geometry. An individual Bi_2_Te_2_Se platelet is excited by circularly polarized laser at perpendicular incidence, when the bias voltage (*V*_sd_) is applied between two Ti/Au contacts. Correspondingly, a local current density *j* flows in the sample, forming a transverse spin current density *j*_s_. f) Spatial map of the photoconductance generated by the circularly polarized light at *V*_sd_ = +1.2 V (left) and *V*_sd_ = −1.2 V (right), respectively. These signals reflect the photoconductance variation Δ*G*_helical_ and are calculated from the equation (top panel), where *σ*+ (*σ*−) corresponds to right (left) circular polarization of the light. Reproduced with permission.^[^ ^215^ ^]^ Copyright 2018, Springer Nature.

In most TIs, electrons in the surface states are spin‐polarized but locked by their momentum.^[^ ^202^, ^203^ ^]^ To probe the spin polarization of TSSs, the prevalent measurements in energy‐momentum space were conducted by spin‐ and angle‐resolved photoemission spectroscopy (spin‐resolved ARPES).^[^ ^201^, ^204^, ^205^, ^206^ ^]^ For instance, Jozwiak et al. reported the manipulation of photoelectron spin polarization in Bi_2_Se_3_ via selection of the light polarization. As can be seen from **Figure** **15**a,b, the spin polarization exhibited strong dependence on the degree of photon polarization and the polarization component (*P_y_*) could be evenly modulated from nearly +1 to −1. Specifically, the mapping of out‐of‐plane spin component (*P*_z_) showed that the photoelectrons were primarily polarized with −0.8 (+0.8) under RCP (LCP) lights accordingly. The authors ascribed the photon‐polarization‐dependent spin‐texture to strong spin–orbit coupling.^[^ ^204^ ^]^ In addition, the LCP/RCP absorptions of the surface states were typically spin‐dependent and different, making the surface spin texture visible under circular dichroism angle‐resolved photoemission spectroscopy.^[^ ^207^, ^208^ ^]^ Therefore, the CD variation in the photoemission intensity was mapped over the full surface band structure of the Bi_2_Se_3_ sample,^[^ ^207^ ^]^ providing a vectorial spin visualization tool to explore SOC materials.

### 7.2 Circular Photogalvanic Effect

Excited by circularly polarized light, the equilibrium of TI system was broken and the spin currents in TSSs were proposed to transform into polarized net electrical currents.^[^ ^209^ ^]^ Due to the spin‐momentum locking of TIs surface states, the coupling between CPL and the electron spin gives a rise of asymmetric distribution of spin‐up and spin‐down carriers, producing CPGE photocurrents that are highly correlated with the helicity and incident angle of excited lights.

As shown in Figure 15c,d, the magnitude of CPGE photocurrent in Bi_2_Se_3_ sample clearly varied with the light polarization and the author ascribed this polarization‐dependent photocurrent to the asymmetric optical excitation of topological and helical Dirac fermions.^[^ ^210^ ^]^ Subsequently, the CPGE in topological insulators was found to be tuned via electrostatic gating, Fermi energy tuning, and spin injection.^[^ ^211^ ^]^ For instance, Okada et al.^[^ ^212^ ^]^ realized the CPGE in (Bi_1−_ *_x_* Sb*_x_*)_2_Te_3_ by changing the Sb component (*x*) to tune the Fermi energy (*E*_F_). When the *E*_F_ was set near the Dirac cone, the CPGE current was remarkably enhanced because of suppressed scattering of the surface‐Dirac electrons to the bulk channel. In addition, by replacing the SrTiO_3_ substrate by Si wafer, the CPGE current of Bi_2_Te_3_ films was also found to be significantly improved by two orders of magnitude, profiting from the extra spin injection from Si substrate to TI.^[^ ^213^ ^]^ However, the surface states observation is often mixed with non‐negligible contribution from the bulk states in conventional transport experiments. To tackle this issue, Kastl et al.^[^ ^214^ ^]^ isolated ultrafast helicity‐dependent currents of TSSs in Bi_2_Se_3_ film from laser heating‐induced thermoelectric currents and drift currents in the bulk on a picosecond timescale by time‐resolved and time‐integrated photocurrent spectroscopy. Until now, the study of CPL‐related circular photogalvanic effect in TIs materials is at the initial stage and more research investments toward this direction may unveil intense feasible opto‐spintronic devices.

### 7.3 Spin Hall Effect

The CPL illumination upon TIs materials with strong spin–orbit coupling typically generates a transverse spin current with the spin polarization perpendicular to the plane, in light of spin Hall effect. Different from traditional Hall effect that charge accumulates along the transversal direction, the spin accumulation in TIs is expected along the sample edges in spin Hall effect. In addition, the inverse spin Hall effect that is the reciprocal process of SHE also exists by conversing the spin into charge for detection. In these effects, the output signals are usually displayed as helicity‐dependent photo‐voltage that reflect the distribution of generated spins. In 2018, Seifert et al.^[^ ^215^ ^]^ reported the opposite spin accumulation at the opposite edges of topological Bi_2_Te_2_Se platelets under normal incidence of circularly polarized laser (Figure 15e). In their experiment, the spatial maps acquired under *V*_bias_ = ±1.2 V exhibited that the photoconductance (Δ*G*) as a consequence of spin accumulation at two lateral edges was opposite (Figure 15f). However, in the center region, the helical conductance (Δ*G*_helical_) approached zero, consistent with the SHE phenomenology. Excited by CPL, similarly, Liu et al.^[^ ^216^ ^]^ reported the direct spatial imaging of current‐induced spin accumulations in Bi_2_Se_3_ and BiSbTeSe_2_ samples even at room temperature. The voltage difference under RCP and LCP excitations exhibited opposite signs along two edges of Bi_2_Se_3_ channel. Moreover, the simple reversion of source–drain current reversed the sign of photovoltages. By applying a magnetic field, the author further demonstrated the spin Hall angles (charge‐to‐spin conversion efficiency) of Bi_2_Se_3_ and BiSbTeSe_2_ to be 0.0085 and 0.0616, respectively. In a nutshell, the implementation of CPL excitation enables the spin Hall effect and inversion spin Hall effect in the absent of magnetic fields, becoming a new doorknob to manipulate electron spins in potential spintronic devices.

## 8 Conclusion and Outlook

In the last decade, intense fascinating physical phenomena were excavated in 2D materials manipulated by circular polarized light. In this review, we discussed the recent process of circularly polarized light–matter interactions in representative 2D materials with distinct constituent structures and properties, including valley polarization, valley Hall effect, optical stark effects, Zeeman effects, and photogalvanic effects. In addition, the energy, polarization, and pulse shape of circularly polarized light become additional doorknobs to tune the electronic and optoelectronic effects in 2DMs in an elegant fashion. With further investments from multidiscipline research fields, the circularly polarized light‐induced phenomena in 2DMs may boost tremendous applications in future optoelectronics, spintronics, and valleytronics. For instance, circularly polarized electroluminescence may find intense applications in TMDs‐based light‐emitting diodes (LEDs) and the selective manipulation of spin‐valley‐coupled photocurrents might be adopted to implement polarization‐sensitive light‐detections and integrated spintronic devices. Circularly polarized femtosecond pulses may induce pseudo‐magnetic field and control valley pseudospin in monolayer TMDs, promising for information storage and communications. Behind these rapid process and glorious vision; however, severe obstacles remain to be addressed before translating the circularized polarized light‐2DM interactions and correlated devices from laboratories into industries.

First, the present 2DMs for light–matter interaction here are mainly obtained via mechanical exfoliation of parent bulk crystals and the dimension of 2DMs usually lies in the range of few to tens of micrometers, limiting the propagation of 2DMs in electronic and optoelectronic applications. Therefore, the massive production of high‐quality and large‐size 2DM samples in a controllable way is highly demanded. In addition, the distribution and density of defects, layer number, stacking order, and twist angle of homo‐ and hetero‐structure rely on intense manpower and complicated procedures. More efforts are required to develop a simple and cost‐effective method to prepare desirable 2DMs toward industrialization.

Second, the observations and manipulations of fascinating phenomenon by circularly polarized light were usually realized in extreme environments such as cryogenic temperature or vacuum conditions. In contrast, the majority of commercial electronic and optoelectronic entities work in atmosphere and at room temperature and even high temperature. Hence, more attention is advocated into pushing the operation limitation before implementation of circularly polarized light–2DM interactions into practical devices.

Last, the effective screenings and predictions of chiral 2DMs by high throughputs calculations are recommended to be conducted to offer valuable guidance for designing high‐performance devices based on circularly polarized light–matter interactions. In several scenarios, the light tunability of figure of merit of 2DMs‐based devices shows the trend of saturation or unsatisfying. Due to non‐invasion and high‐compatibility of light, the multiple couplings and manipulation by additional magnetic and electrical fields may push the exploration of emerging phenomenon in diverse 2DMs to an unprecedented height.

## Conflict of Interest

The authors declare no conflict of interest.
